# IAP inhibitor plus chemoradiotherapy for the treatment of bulky anal canal carcinoma

**DOI:** 10.1007/s12032-023-02224-1

**Published:** 2023-11-14

**Authors:** Francesca De Felice, Pierfrancesco Franco

**Affiliations:** 1 Radiation Oncology, Policlinico Umberto I, Department of Radiological, Oncological and Pathological Sciences, “Sapienza” University of Rome, 00161 Rome, Italy; 2 Department of Translational Medicine (DIMET), Department of Radiation Oncology, “Maggiore della Carità” University Hospital, University of Eastern Piedmont, 28100 Novara, Italy

**Keywords:** Anal canal carcinoma, Squamous cell carcinoma, Definitive radiochemotherapy, Immunotherapy, IAP

## Abstract

The aim of this editorial is to focus on the urgent need to improve clinical outcomes in patients with bulky primary anal canal carcinoma.

## Clinical commentary

Anal canal carcinoma is a relatively rare malignancy with an estimated 50,865 new cases/year and almost 19,300 cancer-related deaths/year globally [1]. Definitive radiotherapy with concurrent mitomycin C and 5-fluoruracil-based chemotherapy (CRT) provides a 5-year overall survival (OS) rate of more than 65% with a colostomy-free survival of approximately 60% and is considered a well-established standard of care worldwide [2]. However, it is associated with persistent disease in 10–15% of patients and a 30% recurrence rate, especially in cases with adverse prognostic factors, such as bulky primary tumor [3]. Generally speaking, primary tumor size has been shown as an independent predictor of disease-free survival and OS in anal cancer patients, as shown in the RTOG 98–11 trial [4]. This finding prompted the American Joint Committee on Cancer (AJCC), in the 8th edition of the TNM staging system, to subdivide stage II squamous cell carcinoma of the anal canal into stage IIA (cT2N0M0) and stage IIB (cT3N0M0), based on primary tumor maximum dimension (≤ 5 versus > 5 cm) [5]. Even more, bulky anal canal carcinoma has a dismal prognosis (OS at 5 years < 40%),] but despite the poor long-term outcomes, its management has remained relatively unchanged for more than 4 decades [3].

Over the years, different treatment strategies have been tested to improve clinical outcomes, but all failed [6, 7]. Novel treatment options are therefore urgently needed in this clinical setting. Currently, the landscape of clinical trials has shifted to immunotherapy. Based on the assumption that anal cancer can be associated to head and neck cancer—mainly because of histology (the vast majority are squamous cell carcinoma), risk factors (most anal canal carcinoma are human papilloma virus (HPV)-related), treatment approaches (definitive CRT) and extended target regions (including therapeutic and prophylactic target volumes)—and considering the recently demonstrated superiority of xevinapant plus CRT in patients with unresected locally advanced squamous cell carcinoma of the head and neck [8], the addition of an inhibitor of inhibitor of apoptosis proteins (IAPs) may be an attractive research hypothesis to be tested in patients with bulky anal canal carcinoma, to potentially improve clinical outcomes. Evaluating IAP inhibitors in this setting of patients could represent a window-of-opportunity for patients with anal canal squamous cell carcinoma.
